# Outcome from 5-year live surgical demonstrations in urinary stone treatment: are outcomes compromised?

**DOI:** 10.1007/s00345-017-2050-4

**Published:** 2017-05-18

**Authors:** Jaap D. Legemate, Stefano P. Zanetti, Joyce Baard, Guido M. Kamphuis, Emanuele Montanari, Olivier Traxer, Jean JMCH de la Rosette

**Affiliations:** 1 Department of Urology, AMC University Hospital, Meibergdreef 9, 1105 AZ Amsterdam Z-O, The Netherlands; 2 Department of Urology, IRCCS Ca’ Granda Ospedale Maggiore Policlinico, University of Milan, Milan, Italy; 3 Department of Urology, Tenon Hospital, Pierre et Marie Curie University, Paris, France

**Keywords:** Stones, Live, Surgical, Demonstrations, Ureteroscopy, Percutaneous, Lithotomy, Urolithiasis

## Abstract

**Purpose:**

To compare intra- and post-operative outcomes of endourological live surgical demonstrations (LSDs) and routine surgical practice (RSP) for urinary stones.

**Methods:**

Consecutive ureterorenoscopic (URS) and percutaneous (PNL) urinary stone procedures over a 5-year period were reviewed. Procedures were divided into LSDs and RSP. Differences between the groups were separately analysed for URS and PNL. Primary outcomes included intra- and post-operative complication rates and grades. Secondary outcomes were operation time, length of hospital stay, stone-free rate, and retreatment rate. Pearson’s Chi-square analysis, Mann–Whitney U test, and logistic and linear regression were used to compare outcomes between LSDs and RSP.

**Results:**

During the study period, we performed 666 URSs and 182 PNLs, and 151 of these procedures were LSDs. Among URSs, the overall intra-operative complication rate was 3.2% for LSDs and 2.5% for RSP (*p* = 0.72) and the overall post-operative complication rate was 13.7% for LSDs and 8.8% for RSP (*p* = 0.13). Among PNLs, the overall intra-operative complication rate was 8.9% for LSDs and 5.6% for RSP (*p* = 0.52) and the overall post-operative complication rate was 28.6% for LSDs and 34.9% for RSP (*p* = 0.40). For both URSs and PNLs, no statistically significant differences in complication grade scores were observed between LSDs and RSP. Operation time was significantly longer for LSD-URS group, but there was no difference between the PNL groups. There were no significant differences in length of hospital stay and stone-free rate. The retreatment rate was higher in the LSD-URS group compared with RSP-URS group but similar between the PNL groups. Multiple logistic regression analyses, adjusting for confounders, revealed no association between LSD and more or less favourable outcomes as compared to RSP.

**Conclusion:**

Live surgical demonstrations do not seem to compromise patients’ safety and outcomes when performed by specialised endourologists.

**Electronic supplementary material:**

The online version of this article (doi:10.1007/s00345-017-2050-4) contains supplementary material, which is available to authorized users.

## Introduction

Live surgical demonstrations (LSDs) are a popular educational tool. With the increased prevalence and popularity of LSDs, their educational value, professional and financial conflicts of interest and, above all, patients’ safety have been questioned [1, 2]. To secure patients’ safety, several surgical societies have published policy statements for the performance of LSDs [3]. The European Association of Urology (EAU) published a policy, which provides supportive information on how to organise and safely conduct LSDs. The paramount principle of this policy is that patients’ safety has top priority over all other considerations [1]. Furthermore, the literature about the safety of LSDs in the urological field is quite scarce. Most of the studies conducted are policy publications or surveys [1, 3–6] and the conclusions of these studies are not based on observations or treatment outcomes.

To obtain more objective information about patients’ safety during LSDs, insight in the surgical outcomes of these procedures is needed. The objective of this study is to evaluate possible differences in outcomes between endourological stone removal procedures performed during LSDs and during routine surgical practice (RSP).

## Material and methods

### Outcomes

Primary outcomes included intra-operative complication rates and post-operative complication rates and Clavien–Dindo grades. Secondary outcomes were operation time, length of hospital stay, stone-free rates, and retreatment rates.

### Data collection, definitions, and setting

Data were retrospectively collected from all consecutive endoscopic procedures performed for urinary stones in the academic center of the leading author from January 2011 through July 2015. Procedures were divided into ureterorenoscopic (URS) and percutaneous procedures. Percutaneous procedures included percutaneous nephrolithotomy (PNL) and endoscopic combined intrarenal surgery (ECIRS). Within these two groups, procedures were divided into cases performed as LSDs and RSP. An LSD was defined as a surgical procedure performed in real time and observed for educational purposes [1]. At our academic hospital, LSDs have been done since 2005. During the study period, we performed 151 LSDs, organized as follows. Visitors participated in a 2-day course. On the first day, lectures were given. The second day visitors watched LSDs via a live broadcast in the hospital or they were present in the operation room. During the surgery, there was a two-way connection to communicate with the surgeon or moderators. Courses had an international setting with participating visitors and surgeons from different countries. The language of communication was English. Surgeons were specialised endourologists from our own institution or guest surgeons with extensive experience in endourology. Guest surgeons were familiar with the equipment and environment of the operating theatres. Surgery performed by a guest surgeon was always accompanied by one or more endourologists from our own institution.

### Study population and selection

Patients included in this study were all patients who underwent endourological stone removal during the study period. During LSDs, a combination of high and low complexity cases was selected, depending on the available patients. Patients operated in LSDs were well informed about the theatre setting and guest surgeons. All patients signed an informed consent.

### Data collection

Patient’s demographic and clinical characteristics were assessed. Total stone burden was computed as the stone surface area (SSA) on imaging with the formula: length*width*0.25*π [7, 8].

The SSA was computed on the CT scan transverse slice in which the stone appeared the widest. In case of multiple stones, the sum of the single SSAs was computed.

#### Outcomes and follow-up

Intra-operative complications included: bleeding, perforation, ureteral damage, avulsion, and complications outside the urinary tract. Post-operative complications were: bleeding, fever (>38.0 °C), urinary tract infection, sepsis, pain needing intervention, urinary leakage, death, and complications outside the urinary tract. Post-operative complications were classified according to the Clavien–Dindo classification [9]. For PCNL, the validated Clavien score for percutaneous nephrolithotomy was used [10].

Failure of the procedure was defined as failed access to the ureter or the collecting system, leaving the stone in situ. Operation time was defined as the time interval between the introduction and the extraction of the scope for URS and from the puncture to the positioning of the nephrostomy for PNLs. In case of tubeless PNLs, the extraction of the scope and the Amplatz sheath was considered the end of the procedure. The total follow-up period was 3 months. The stone-free status was assessed with ultrasound or computed tomography at 6–8 weeks after the procedure. Stone-free was defined as the absence of fragments >1 mm on CT or total absence of fragments on ultrasound. In the absence of post-operative imaging, intra-operative confirmation with the absence of stones >1 mm was applied to meet a stone-free status.

### Statistical analysis

Comparative analysis was performed using Pearson’s Chi-square or Fisher’s exact test for categorical variables and Mann–Whitney U test for not normally distributed continuous variables. Multiple linear and logistic regression analyses were performed to analyse the association between LSD and outcomes as they differed from RSP. Outcomes were adjusted for confounders. The confounding variables for URS were stone burden, renal anomalies, stone location. A confounding factor for PNL was the stone burden. For all analyses, the level of statistical significance was set at *p* < 0.05. All calculations were performed using IBM SPSS 23.0.

## Results

### Patient and procedure characteristics

We included 666 URS and 182 percutaneous procedures. In the URS group, 95 (14.3%) procedures were LSDs. In the percutaneous procedures group, 56 (30.8%) were LSDs. Tables 1 and 2 present baseline characteristics of patients operated during LSDs and RSP, which were well matched for both groups, with the exception of the amount of renal anomalies which was significantly higher in the LSD-URS group as compared with the RSP-URS group.

**Table 1:** Baseline characteristics of ureteroscopy for stones

Characteristics	All procedures *n* = 666	LSD-URS *n* = 95 (14.3)	RSP-URS *n* = 571 (85.7)	*p* value	Test
Age, median in years [IQR]	53, [40–65]	53, [39–61]	53, [40–65]	0.56	C
	(*n* = 666)	(*n* = 95)	(*n* = 571)		
Gender					
Male, *n* (%)	423 (63.5)	57 (60.0)	366 (64.1)	0.44	A
Female, *n* (%)	243 (36.5)	38 (40.0)	205 (35.9)		
	(*n* = 666)	(*n* = 95)	(*n* = 571)		
BMI median [IQR]	26.0, [23.8–9.0]	25.9, [24.2–29.6]	26.0, [23.7–29.0]	0.57	C
	(*n* = 652)	(*n* = 91)	(*n* = 561)		
ASA score, *n* (%)					
I	242 (36.3)	37 (38.9)	205 (35.9)	0.85	A
II	336 (50.5)	45 (47.4)	291 (51.0)		
III	86 (12.9)	13 (13.7)	73 (12.8)		
IV	2 (0.3)	0 (0)	2 (0.4)		
	(*n* = 666)	(*n* = 95)	(*n* = 571)		
Comorbidity and medication, *n* (%)					
DM	90 (13.5)	16 (17.8)	74 (13.0)	0.31	A
	(*n* = 665)	(*n* = 95)	(*n* = 570)		
CVD	245 (36.8)	30 (31.6)	215 (37.7)	0.25	A
	(*n* = 665)	(*n* = 95)	(*n* = 570)		
Crohn’s disease	20 (3.0)	0 (0)	20 (3.4)	0.096	B
	(*n* = 665)	(*n* = 95)	(*n* = 570)		
Prednisone	30 (4.5)	4 (4.2)	26 (4.6)	1.0	B
	(*n* = 665)	(*n* = 95)	(*n* = 570)		
Anticoagulation	117 (17.6)	11 (9.4)	106 (18.6)	0.096	A
	(*n* = 665)	(*n* = 95)	(*n* = 570)		
Previous stone treatment in the same renal unit, *n* (%)					
URS	253 (38.0)	43 (45.3)	210 (36.8)	0.12	A
	(*n* = 666)	(*n* = 95)	(*n* = 571)		
PCNL	87 (13.1)	20 (21.1)	67 (11.7)	0.013	A
	(*n* = 666)	(*n* = 95)	(*n* = 686)		
SWL	123 (15.9)	20 (19.6)	82 (14.4)	0.087	A
	(*n* = 663)	(*n* = 95)	(*n* = 569)		
Ureterolithotomy	6 (0.9)	0 (0)	6 (1.1)	0.60	B
	(*n* = 666)	(*n* = 95)	(*n* = 571)		
Pyelolithomy	8 (1.2)	4 (4.2)	4 (0.7)	0.017	B
	(*n* = 665)	(*n* = 95)	(*n* = 570)		
Anatomical variant					
Anomalies	37 (5.6)	11 (11.6)	26 (4.6)	0.006	A
	(*n* = 666)	(*n* = 95)	(*n* = 571)		
Solitary kidney	16 (2.4)	2 (2.1)	14 (2.5)	1.00	B
	(*n* = 666)	(*n* = 95)	(*n* = 571)		

Statistical tests: A) Pearson’s Chi-square test, B) Fisher’s exact test, and C) Mann–Whitney U test. Data are *n* (%) of patients for whom data were available. Percentages exclude missing values from denominators

*ASA* American Society of Anesthesiologists, *DM* diabetes mellitus, *CVD* cardio vascular disease, *PCNL* percutaneous nephrolithotomy, *ESWL* extra corporeal shockwave lithotripsy, *URS* ureterorenoscopy, *UPJ* uretero-pelvic junction, *NS* not significant

**Table 2:** Baseline characteristics of percutaneous stone procedures

Characteristics	All procedures *n* = 182	LSD percutaneous *n* = 56 (30.8)	RSP percutaneous *n* = 126 (69.2)	*p* value	Test
Age, median in years [IQR]	51, [36–66]	53.5, [38–67]	51, [34–65]	0.30	C
	(*n* = 182)	(*n* = 56)	(*n* = 126)		
Gender					
Male, *n* (%)	107 (58.8)	29 (51.8)	78 (61.9)	0.20	A
Female, *n* (%)	75 (41.2)	27 (48.2)	48 (38.1)		
	(*n* = 182)	(*n* = 56)	(*n* = 126)		
BMI, median [IQR]	25.6, [22.8–28.2]	25.1, [22.4–27.9]	25.6, [23.1–28.4]	0.43	C
	(*n* = 179)	(*n* = 56)	(*n* = 123)		
ASA score, *n* (%)					
I	55 (30.2)	19 (33.9)	36 (28.6)	0.39	A
II	101 (55.5)	27 (48.2)	74 (58.7)		
III	26 (14.3)	10 (17.9)	16 (12.7)		
IV	0 (0)	0 (0)	0 (0)		
	(*n* = 182)	(*n* = 56)	(*n* = 126)		
Comorbidity and medication, *n* (%)					
DM	18 (9.9)	5 (8.9)	13 (10.3)	1.00	B
	(*n* = 182)	(*n* = 56)	(*n* = 126)		
CVD	79 (43.4)	23 (41.4)	56 (44.4)	0.67	A
	(*n* = 126)	(*n* = 56)	(*n* = 126)		
Crohn’s disease	3 (1.6)	0 (0)	3 (2.4)	0.55	B
	(*n* = 126)	(*n* = 56)	(*n* = 126)		
Prednisone	14 (7.7)	2 (3.6)	12 (9.5)	0.23	B
	(*n* = 126)	(*n* = 56)	(*n* = 126)		
Anticoagulation	29 (15.9)	11 (19.6)	18 (14.3)	0.36	A
	(*n* = 128)	(*n* = 56)	(*n* = 126)		
Previous stone treatment, *n* (%)	107 (58.8)	32 (57.1)	75 (59.5)	0.76	A
In the same renal unit	(*n* = 182)	(*n* = 56)	(*n* = 126)		
SWL	37 (20.3)	12 (21.4)	25 (19.8)	0.81	A
	(*n* = 182)	(*n* = 56)	(*n* = 126)		
URS	33 (18.1)	12 (21.4)	21 (16.7)	0.44	A
	(*n* = 182)	(*n* = 56)	(*n* = 126)		
PCNL	55 (30.2)	13 (23.2)	42 (33.3)	0.17	A
	(*n* = 182)	(*n* = 56)	(*n* = 126)		
ECIRS	24 (13.2)	10 (17.9)	14 (11.1)	0.24	B
	(*n* = 182)	(*n* = 56)	(*n* = 126)		
Pyelolithomy	9 (4.9)	3 (5.4)	6 (4.8)	1.00	B
	(*n* = 182)	(*n* = 56)	(*n* = 126)		
Anatomical variant					
Renal anomalies, *n* (%)	9 (4.9)	4 (7.1)	5 (4.0)	0.46	B
	(*n* = 182)	(*n* = 56)	(*n* = 126)		
Solitary kidney, *n* (%)	5 (2.7)	2 (3.6)	3 (2.4)	0.64	B
	(*n* = 182)	(*n* = 56)	(*n* = 126)		

Statistical test: A) Pearson’s Chi-square test, B) Fisher’s exact test, and C) Mann–Whitney U test. Data are *n* (%) of patients for whom data were available. Percentages exclude missing values from denominators

*ASA* American Society of Anesthesiologists, *DM* diabetes mellitus, *CVD* cardio vascular disease, *PCNL* percutaneous nephrolithotomy, *ESWL* extra corporeal shockwave lithotripsy, *URS* ureterorenoscopy, *NS* not significant

Tables 3 and 4 present pre-operative clinical information. For URS, the median SSA was 91 mm^2^ in the LSDs group and 48 mm^2^ in the RSP group (*p* < 0.001). In LSDs, the stone was located in the kidney in 84.2%, while in RSP in 49.7% (*p* < 0.001) of the cases. There was no significant difference in pre- and post-operative ureteral stent placement.

**Table 3:** Operation data of ureteroscopy for stones

Outcomes	LSD-URS *n* = 95 (14.3)	RSP-URS *n* = 571 (85.7)	*p* value	Test
Total stone burden (mm^2^) median,[IQR]	91, [44–192]	48, [25–87]	0.001	C
	(*n* = 95)	(*n* = 558)		
General stone location				
Kidney	80 (84.2)	284 (49.7)	0.001	A
Ureter	11 (11.6)	235 (41.2)		
Kidney and ureter	4 (4.2)	52 (9.1)		
	(*n* = 95)	(*n* = 571)		
Kidney stone location, *n* (%)				
Renal pelvis	12 (15.0)	48 (16.9)	0.93	A
Upper calix	5 (6.3)	17 (6.0)		
Middle calix	8 (10.0)	26 (9.2)		
Lower calix	26 (32.5)	103 (36.3)		
Multiple	29 (36.3)	90 (31.7)		
	(*n* = 80)	(*n* = 284)		
Ureteral location, *n* (%)				
Proximal	3 (27.3)	83 (35.3)	0.25	A
Mid	2 (18.2)	45 (19.1)		
Distal	5 (45.5)	104 (44.3)		
Multiple	1 (9.1)	3 (1.3)		
	(*n* = 11)	(*n* = 235)		
Pre-operative ureteral stent, *n* (%)	28 (29.5)	174 (30.5)	0.84	A
	(*n* = 95)	(*n* = 571)		
Exit strategy				
Double J stent	52 (54.7)	283 (49.6)	0.19	A
Ureteral catheter	27 (28.4)	156 (27.3)		
Nephrostomy tube	1 (1.1)	10 (1.8)		
Ureteral stent and nephrostomy	5 (5.3)	14 (2.5)		
Tubeless	10 (10.5)	108 (18.9)		
	(*n* = 95)	(*n* = 571)		

Statistical tests: A) Pearson’s Chi-square test, B) Fisher’s exact test, and C) Mann–Whitney U test

* Nephrostomy tube and a double J stent or ureteral catheter. Data are *n* (%) of patients for whom data were available. Percentages exclude missing values from denominators

**Table 4:** Surgical data of percutaneous stone procedures

Outcomes	LSD percutaneous *n* = 56 (30.8)	RSP percutaneous *n* = 126 (69.2)	*p* value	Test
Total stone burden (mm^2^) median, [IQR]	389, [228–676]	280, [132–595]	0.039	C
	(*n* = 54)	(*n* = 107)		
Total stone diameter, median in mm, [IQR]	41 [27–66]	28 [18–68]	0.063	C
	(*n* = 54)	(*n* = 108)		
Density in hounsfield units, median, [IQR]	950, [678–1189]	900, [661–1020]	0.22	C
	(*n* = 45)	(*n* = 101)		
Stone location, *n* (%)				
Renal pelvis	10 (17.9)	17 (13.5)	0.14	A
Upper calix	0 (0)	8 (6.3)		
Middle calix	1 (1.8)	2 (1.6)		
Lower calix	8 (14.3)	21 (16.7)		
Staghorn stone	8 (14.3)	14 (11.1)		
Ureteral stone	0 (0)	10 (7.9)		
Multiple locations*	29 (51.8)	54 (42.9)		
	(*n* = 56)	(*n* = 126)		
Pre-operative ureteral stent *n* (%)	9 (16.1)	19 (15.1)	0.83	B
	(*n* = 56)	(*n* = 126)		
Pre-operative percutaneous drain *n* (%)	6 (10.7)	27 (21.4)	0.098	B
	(*n* = 56)	(*n* = 126)		
Exit strategy				
Ureteral stent**	5 (8.9)	8 (6.5)	0.31	A
Nephrostomy tube	28 (50.0)	78 (63.4)		
Ureteral stent and nephrostomy	23 (41.1)	36 (29.3)		
Tubeless	0 (0)	1 (0.8)		
	(*n* = 56)	(*n* = 123)		
Type of operation				
PNL only***	9 (16.1)	82 (65.1)	0.001	B
ECIRS	47 (83.9)	44 (34.9)		
	(*n* = 56)	(*n* = 126)		

Statistical test: A) Pearson’s Chi-square test, B) Fisher’s exact test, and C) Mann–Whitney U test. Data are *n* (%) of patients for whom data were available. Percentages exclude missing values from denominators

*ECIRS* endoscopic combined intrarenal surgery

* Ureter included

** Including ureteral stent and double J catheter

*** Including prone and supine

In the percutaneous procedures group, the average SSA was 389 mm^2^ for LSDs and 280 mm^2^ for RSP (*p* = 0.0.39). Patients had stones in multiple anatomical locations of the kidney or ureter in 51.8% of the LSDs and in 42.9% of the RSP procedures. An ECIRS was performed in 83.9% of the LSDs and in 34.9% of the RSP procedures (*p* < 0.001).

### Ureteroscopic procedures

Descriptive data on complications and secondary outcomes for URS procedures are presented in Table 5. There were no significant differences either in the overall intra-operative complication rates (3.2% for LSDs vs. 2.5% for RSP, *p* = 0.72) or in the overall post-operative complication rates (13.7% for LSDs vs. 8.8% for RSP, *p* = 0.13). The majority of the complications were classified as Clavien 2 in both groups. In both groups, one Clavien 5 complication occurred.

**Table 5:** Intra- and post-operative complications and outcomes of ureteroscopy for stones

Outcomes	LSD-URS *n* = 95 (14.3)	RSP-URS *n* = 571 (85.7)	*p* value	Test
Intra-operative complications, *n* (%)				
Overall	3 (3.2)	14 (2.5)	0.72	B
Bleeding	1 (1.1)	3 (0.5)	0.46	B
Perforation	0 (0)	10 (1.8)	0.37	B
Avulsion	0 (0)	0 (0)		
Other	2 (2.1)	1 (0.2)	0.055	B
	(*n* = 95)	(*n* = 571)		
Post-operative complications, *n* (%)				
Overall*	13 (13.7)	50 (8.8)	0.13	A
Bleeding	0 (0)	3 (0.5)	1.00	B
Fever (>38.0)	7 (7.4)	19 (3.3)	0.080	B
UTI	5 (5.3)	17 (3.0)	0.23	B
Sepsis	4 (4.2)	16 (2.8)	0.51	B
Pain	0 (0)	13 (2.3)	0.23	B
Death	1 (1.1)	1 (0.2)	0.27	B
Other	2 (2.1)	1 (0.2)	0.21	B
	(*n* = 95)	(*n* = 571)		
Total blood transfusion rate, *n* (%)	0 (0)	6 (1.1)	1.00	B
	(*n* = 94)	(*n* = 571)		
Clavien grading score, *n* (%)				
None	82 (86.3)	521 (91.2)	0.12	A
1	0 (0)	6 (1.1)		
2	11 (11.6)	31 (5.4)		
3a	0 (0)	6 (1.1)		
3b	0 (0)	3 (0.5)		
4a	1 (1.1)	3 (0.5)		
4b	0 (0)	0 (0)		
5	1 (1.1)	1 (0.2)		
	(*n* = 95)	(*n* = 571)		
Failed procedures	8 (8.4)	30 (5.3)	0.23	B
	(*n* = 95)	(*n* = 571)		
Operation time minutes, median, [IQR]	50, [40.5–68]	41, [25–63]	<0.001	C
	(*n* = 95)	(*n* = 570)		
Post-operative length of hospital stay	1.0, [1.0–1.0]	1.0, [1.0–1.0]	0.83	C
days, median, [IQR]	(*n* = 94)	(*n* = 571)		
Stone-free rate	58 (63.0)	366 (67.7)	0.39	A
	(*n* = 92)	(*n* = 541)		
Method of evaluation				
Computed tomography	6 (6.3)	20 (3.5)	0.19	A
Ultrasound	56 (58.9)	430 (75.3)	<0.001	A
Intra-operative confirmation	26 (27.4)	106 (18.6)	0.046	A
Other	7 (7.4)	15 (2.6)	0.017	A
	(*n* = 95)	(*n* = 571)		
Retreatment, *n* (%)	18 (18.9)	64 (11.2)	0.034	A
	(*n* = 95)	(*n* = 571)		
Readmission <3 months, *n* (%)	19 (20.0)	76 (13.4)	0.088	A
	(*n* = 95)	(*n* = 568)		

*UTI* urinary tract infection, *KUB* kidneys, ureters, bladder

* Number of procedures with post-operative complications. Per procedure more than one complication could have been scored. Statistical tests: A) Pearson’s Chi-square test, B) Fisher’s exact test, and C) Mann–Whitney U test. Data are *n* (%) of patients for whom data were available. Percentages exclude missing values from denominators

The median operation time was 50 min in the LSD group and 41 min in the RSP group (*p* < 0.001). The median length of hospital stay was 1 day in both groups. The stone-free rate was 63.0% in the LSD group and 67.7% in the RSP group (*p* = 0.39). A retreatment was needed in 18.9% of LSDs and 11.2% of RSP procedures (*p* = 0.034).

### Percutaneous procedures

Descriptive data on complications and secondary outcomes for percutaneous procedures are presented in Table 6.

**Table 6:** Intra- and post-operative complications and outcomes of the following percutaneous stone procedures

Outcomes	LSD percutaneous *n* = 56 (30.8)	RSP percutaneous *n* = 126 (69.2)	*p* value	Test
Intra-operative complications, *n* (%)				
Overall	5 (8.9)	7 (5.6)	0.52	B
Bleeding*	3 (5.4)	4 (3.2)	0.68	B
Ureteral perforation	0 (0)	2 (1.6)	1.0	B
Mucosal damage of the ureter	1 (1.8)	1 (0.8)	0.52	B
Ureteral avulsion	0 (0)	0 (0)		
Liver puncture	1 (1.8)	0 (0)	0.31	B
Other	0 (0)	0 (0)		
	(*n* = 56)	(*n* = 126)		
Post-operative complications, *n* (%)				
Overall	16 (28.6)	44 (34.9)	0.40	A
Bleeding	3 (5.4)	15 (11.9)	0.28	B
Fever (>38.0)	4 (7.1)	8 (6.3)	1.00	B
UTI	6 (10.7)	7 (5.6)	0.22	B
Sepsis	1 (1.8)	8 (6.3)	0.29	B
urinary leakage	3 (5.4)	2 (1.6)	0.17	B
Death	0 (0)	1 (0.8)	1.00	B
Other	2 (3.6)	10 (7.9)	0.35	B
	(*n* = 56)	(*n* = 126)		
Blood transfusion rate, *n* (%)	3 (5.4)	9 (7.1)	0.76	B
	(*n* = 56)	(*n* = 126)		
Clavien grading score, *n* (%)				
None	40 (71.4)	82 (65.1)	0.51	A
1	1 (1.8)	3 (2.4)		
2	11 (19.6)	18 (14.3)		
3a	2 (3.6)	14 (11.0)		
3b	2 (3.6)	5 (4.0)		
4a	0 (0)	3 (2.4)		
4b	0 (0)	0 (0)		
5	0 (0)	1 (0.8)		
	(*n* = 56)	(*n* = 126)		
Failed procedures	1 (1.8)	8 (6.4)	0.28	B
	(*n* = 56)	(*n* = 125)		
Operation time (minutes), median, [IQR]	79.5, [62–101.5]	75, [57–94]	0.21	C
	(*n* = 56)	(*n* = 125)		
Post-operative length of hospital stay (days), median, [IQR]	4, [3–5]	3, [2–4]	0.086	C
	(*n* = 56)	(*n* = 119)		
Stone-free rate in follow-up	26 (50.0)	45 (38.5)	0.16	A
	(*n* = 52)	(*n* = 117)		
Method of evaluation				
Computed tomography	11 (21.6)	20 (17.5)	0.33	A
Ultrasound	35 (68.6)	70 (61.4)		
Retrograde pyelogram	0 (0)	2 (1.8)		
Intra-operative confirmation*	5 (9.8)	22 (19.3)		
	(*n* = 51)	(*n* = 114)		
Time until follow-up moment, weeks, median [IQR]	8 [12]	6 [4]	0.008	C
	(*n* = 46)	(*n* = 91)		
Retreatment, *n* (%)	14 (25.5)	45 (36.0)	0.16	A
	(*n* = 55)	(*n* = 124)		
Readmission <3 months, *n* (%)	18 (32.1)	47 (37.6)	0.48	A
	(*n* = 56)	(*n* = 125)		

*UTI* urinary tract infection

* No intra-operative blood transfusions were needed. Statistical test: A) Pearson’s Chi-square test, B) Fisher’s exact test, and C) Mann–Whitney U test. Data are *n* (%) of patients for whom data were available. Percentages exclude missing values from denominators

There were neither significant differences in the overall intra-operative complication rates (8.9% for LSDs vs. 5.6% for RSP, *p* = 0.052) nor in the overall post-operative complication rates (28.6% for LSDs vs. 34.9% for RSP, *p* = 0.40). There were no significant differences in Clavien complication grades between the groups. One Clavien 5 complication occurred in the RSP group.

Operation time did not significantly differ between LSDs and RSP. The median length of hospital stay was 4 days after LSDs and 3 days after RSP procedures (*p* = 0.086). The stone-free rate was 50.0% in the LSD group and 38.5% in the RSP group (*p* = 0.16). After LSDs, 25.5% of the patients required retreatment compared with 36.0% after RSP procedures (*p* = 0.16).

### Regression models

For both URS and PNL, the model revealed no association between the factors in LSDs and higher intra- and post-operative complications, longer hospital stay, lower stone-free rates, higher retreatment rates, and longer operation times. The complete univariate and multivariate models can be found in Table S1 of the supplementary material.

## Discussion

The most important finding of this study is that we did not observe major statistically significant differences either in overall intra-operative or in post-operative complication rates and grades between LSDs and RSP for urinary stones.

We observed higher complication rates for LSDs then for RSP in the URS group, but the difference is not statistically significant and can be explained by external factors. The median stone burden in LSDs was almost twice as large as in RSP. The regression model supports that this has contributed to the longer operation time of LSDs. Furthermore, in the LSD group, the percentage of kidney stones was higher than in the RSP group and complication rates in URS are higher in case of renal stones than of ureteral stones [11, 12]. In addition, the percentage of patients with renal anomalies was higher in the LSD group. The presence of a renal anomalies was previously be found to be a factor affecting complication rates [13].

The majority of post-operative complications in both URS- and PNL-LSD groups were classified as Clavien 1 or 2 and no invasive intervention was needed. However, in both the URS-LSD and the URS-RSP groups, one Clavien 5 complication occurred. In the LSD group, this concerned an 85-year-old patient who was in a poor pre-operative condition. The patient was admitted with an obstructive distal ureteral stone that caused urinary tract infection, hydronephrosis, and severe delirium. A percutaneous renal drain was placed and antimicrobial treatment was started. After admittance for 22 days in the hospital, an uncomplicated URS was performed. The post-operative course was complicated by autonomic dysregulation without a plausible reason. 26 days after surgery, an aspiration pneumonia with further deterioration of clinical conditions led to the decision to follow a palliative setting. In this study, the characteristics of patients operated during LSDs and RSP were comparable. Therefore, patients selected for LSDs are representative of the usual cases we treat in our stone referral centre. In this particular case, we do not feel that we could have prevented these complications by operating this patient during RSP. Due to the relatively high frequency of LSDs in our centre, it happens that also more fragile patients are operated on LSD days. In some cases, patients are scheduled in a semi-emergency setting during LSD. This way, we also shorten their waiting time for surgery. In this described case, the patient was admitted for a long period and was being worked up for surgery.

In the RSP group. a Clavien V complication occurred in a 88-year-old ASA 3 patient with a medical history a hypophysis tumour, multiple CVAs with a left sided hemiparesis, renal insufficiency due to hypertension and ischemia after an acute myocardial infarction which, together with a severe aortic valve stenosis, resulted in a very poor cardiac function. After the complete resolution of a urosepsis due to an obstructive ureteral stone, a URS with stone disintegration was performed. Despite perioperative antibiotic prophylaxis, based on previous cultures, the patient post-operatively developed a urosepsis with cardiac decompensation. Support of intensive care initially attained sufficient clinical progression. However, after another flair of urosepsis with severe cardiac decompensation in an elderly highly morbid patient, it was decided to follow a palliative setting 20 days after the surgery.

The mortality rate of 2:666 cases in this cohort is high. In both cases, the pros and cons of alternative options for URS were balanced accurately in a team of staff members and with the patients. These cases reflect the highly complex situations we sometimes face, in which, unfortunately, severe complications can occur.

We observed some differences between the LSD and the RSP groups for what concerns the secondary outcomes of this study. The longer operation time and the higher retreatment rate for URS-LSDs can be explained by the larger median stone burden compared to RSP procedures. Moreover, LSDs represent educational moments and the surgeon is expected to explain and respond to comments. This may prolong the operation time too. Multitasking skills are required to perform an educational but safe and effective surgery avoiding unnecessary extension of the operation time.

The higher stone-free rate after PNL-LSDs may be due to the higher rate of ECIRS performed in this setting compared with RSP. In LSDs, there is often the availability to utilize two surgeons who can perform a simultaneous antegrade and retrograde approach which could result in higher stone-free rates [14]. An explanation for the higher use of ECIRS during LSDs is the higher prevalence of patients with stones in multiple locations and the higher median stone burden.

In this study, the stone-free rates were rather unsatisfactory for both PNL and URS groups. A possible explanation is our strict definition of stone-free status with a cutoff of residual fragments <1 mm. Second, the portion of failed procedures, which was 5.7% in the total URS group, is also included in this calculation. Third, we prefer to approach large stones in staged procedures to limit operation time and thereby the possible occurrence of complications. Finally, training and teaching situations could have had a negative impact on effectiveness in both LSD and RSP settings.

In conclusion, differences in outcomes seem to be explainable and the outcomes in the regression models, adjusted for differences between groups, were not associated with less favourable but also not to more favourable outcomes for LSDs.

Despite the fact that none of the differences between outcomes were statistically significant, we observed certain trends. For URS-LSD, there was a trend towards less favourable outcomes for what concerns intra- and post-operative complications, stone-free rates, operation time, and retreatment rates. After adjustment for confounders, this trend was confirmed for post-operative complications. For PNL-LSD, there was a trend towards less favourable outcomes for what concerns intra-operative complications and operation time. After adjustment for confounders, this trend was confirmed for intra-operative complications. We cannot deny these trends and we think that careful monitoring of future outcomes is a paramount consideration.

### Theoretical framework

To our knowledge, there are no studies comparing the outcomes of endourological procedures performed during LSDs and RSP. In the field of urology, a study by Mullins et al. compared outcomes of 39 robot-assisted partial nephrectomy procedures performed as LSDs with 847 procedures performed as RSP. They found no significant differences in operation time, warm ischemia time, positivity of surgical margin rates, and complication rates. The authors conclude that live robotic surgery can be performed safely [15].

In the field of gastroenterology, outcomes of endoscopic retrograde cholangiopancreatography (ERCP) performed as live demonstrations were compared with routine procedures. Liao et al. performed a large multicentre study including 406 patients that were matched with control patients. The overall complication rate of ERCP in LSDs was not significantly different from RSP. They observed a small but statistically significant difference in treatment success rates unfavourable to LSDs (94.1% vs. 97.5%, *p* = 0.021). Treatment success and complications after ERCP were similar if the procedures were performed by local faculty, domestic visiting, and foreign visiting faculties [16].

With respect to complications rates, the findings of the above-mentioned studies are in agreement with the findings of this study.

Several groups evaluated patients’ safety, educational value and ethical issues surrounding LSDs with surveys. Khan et al. demonstrated higher anxiety levels in surgeons operating in a foreign environment. Difficult communication in the operative theatre, equipment issues, and travel-fatigue appeared to have impact on the surgical performance [4]. Duty et al. performed a survey investigating 90 American Association of Genitourinary Surgeons members. In this study, 28.2% felt moderate, 9.9% high, and 8.5% very high anxiety levels while performing LSDs in their home institution. Visiting physicians’ anxiety levels were rated moderate by 29.8%, high by 25.0%, and very high by 17.9% of the respondents. Noteworthy, only 28.2% of the responders would let a visiting colleague operate on themselves or on a family member [3].

A tool that may contribute to patients’ safety is the more frequent use of ‘as live surgery’ (ALS) instead of LSDs. During ALS, the host and beholders can comment and debate ‘as live’ on a pre-recorded operation.

A survey study by Finch et al. was conducted on 165 participants and evaluated patients’ safety, educational value, and ethical issues of ALS versus LSDs. Participants felt that there were significant patients’ safety benefits with ALS over LSDs. In the same study, participants were significantly less likely to recommend participation in LSDs to family members or friends. Subsequently, they were significantly less likely to participate in an LSD setting themselves [17]. A survey study by Phan et al. concluded that the educational value of ALS is not inferior to LSD broadcasts. The majority of participants agreed that prior LSDs they watched were performed safely [6].

When organizing a live surgery event, organizers and participating physicians should adhere to a clearly defined regulatory framework. The ‘EAU live surgery code of conduct’ can contribute to the organisation of a safe event [1]. A final important aspect is patients’ safety. This does not only concern the physical safety during and after the surgery, but also the respect of patients’ autonomy and privacy. Social media are an increasingly used medium to share knowledge. Regularly, patient’s image material is being shared. Even though the material is not traceable to the patient’s identity, it could contain information that the patient did not give permission to share on the World Wide Web. Everyone participating in LSDs should be aware of this and respect patients’ privacy.

This study supports the assumption that endourological stone treatment during LSDs does not compromise patients’ safety. Still, caution should be exercised drawing a firm conclusion.

A limitation of this study is its retrospective design, which might have led to underestimating the complication rates. Furthermore, this is a single centre study with a restricted group of surgeons operating in a center that organized many LSD events in the studied years, making a routine of it for the local staff.

Even though it was not the objective of the present project, it could be interesting to verify whether there is a difference in outcomes between local and visiting surgeons. In many of the procedures in this study, both a local and a visiting surgeon participated, e.g., ECIRS, so a subanalysis with adequate statistical power was not implemented in this report. The majority of the procedures included in this study were performed by two experienced endourologists: one local and one visiting surgeon. Visiting surgeons were in the vast majority of the cases familiar with the circumstances, equipment and environment during LSDs. For this reason, the findings of this study may not directly assert for LSDs during larger conferences with a larger number of visiting surgeons.

## Conclusion

The findings of this study indicate that live surgical demonstrations for endourological stone removal do not compromise patients’ safety and outcomes when performed by specialised endourologists in a familiar setting. Either way, when performing LSDs, careful monitoring of outcomes is a paramount consideration.

## Electronic supplementary material

Below is the link to the electronic supplementary material.

Supplementary material 1 (DOCX 20 kb)

## Abbreviations

ALS: As live surgery
ECIRS: Endoscopic combined intrarenal surgery
LSD: Live surgical demonstration
RSP: Routine surgical practice
SSA: Stone surface area
