# Supplementary material for: Outcome from 5-year live surgical demonstrations in urinary stone treatment: are outcomes compromised?
Source: World J Urol. 2017 May 18;35(11):1745–56. doi: 10.1007/s00345-017-2050-4 (PMC5649595; doi:10.1007/s00345-017-2050-4)
Supplement: Supplementary file 1 — Supplementary material 1 (DOCX 20 kb) [file 345_2017_2050_MOESM1_ESM.docx]

**Supplementary material**

Table 2. Multiple logistic and linear regression models for the association of live surgical demonstrations with outcomes.

| URS | univariate coeff [CI] | p-value | Multiple regression coeff [CI] | p-value |
| --- | --- | --- | --- | --- |
| Intra-operative complications | -0.260 [0.22-2.74] | 0.687 | 0.029 [0.26-4.03] | 0.967 |
| Postoperative complications | 0.502 [0.86-3.17] | 0.132 | 0.418 [0.75-3.06] | 0.242 |
| High grade complications  >Clavien grade II | -0.659 [0.10-2.65] | 0.429 | -0.186 [0.15-4.74] | 0.830 |
| Length of hospital stay | -0.137 [0.50-1.51] | 0.762 | - | - |
| Stone-free rates | -0.204 [0.52-1.29] | 0.385 | 0.414 [0.90-2.55] | 0.119 |
| Retreatment rates | 0.634 [1.06-3.35] | 0.031 | 0.087 [0.57-2.09] | 0.793 |
| Operation time* | 0.241 [0.11-0.38] | <0.001 | -0.006 [-0.13-0.12] | 0.923 |
| PNL | **univariate coeff [CI]** | **p-value** | **Multiple regression coeff [CI]** | **p-value** |
| Intra-operative complications | 0.511 [0.51-5.50] | 0.402 | 0.666 [0.53-7.15] | 0.316 |
| Postoperative complications | -0.294 [0.38-1.48] | 0.401 | -0.456 [0.30-1.32] | 0.225 |
| High grade complications  >Clavien grade II | -1.190 [0.085-1.09] | 0.068 | -0.843 [0.11-1.73] | 0.234 |
| Length of hospital stay | 0.127 [0.55-2.34] | 0.731 | - | - |
| Stone-free rates | 0.470 [0.83-3.09] | 0.162 | 0.297 [0.66-2.73] | 0.410 |
| Retreatment rates | -0.512 [0.295-1.218] | 0.157 | -0.586 [0.26-1.20] | 0.135 |
| Operation time* | 0.070 [-0.061-0.20] | 0.292 | 0.052 [-0.077-0.18] | 0.428 |

coeff: regression coefficient, CI: 95% confidence interval. Length of hospital stay is not adjusted for confounders in a multiple linear regression analysis. The URS group variables were adjusted for renal anomalies, stone burden and difference in stone location. The PNL group variables were adjusted for stone burden. *logistic transformation. Length of hospital stay was dichotomized for URS longer or shorter than one day and for PNL longer or shorter than four days.

**Additional information**

Article title: Outcome from five-year Live Surgical Demonstrations in urinary stone treatment:

are outcomes compromised?

Journal name: World Journal of Urology

Author names: Jaap D Legemate^a,^*^,§^, Stefano P Zanetti^b^*, Joyce Baard^a^, Guido M Kamphuis^a^, Emanuele Montanari^b^, Olivier Traxer^c^, Jean JMCH de la Rosette^a^

^a^Department of Urology, AMC University Hospital, Amsterdam, the Netherlands

^b^Department of Urology, IRCCS Ca’ Granda Ospedale Maggiore Policlinico, University of Milan, Italy

^c^Department of Urology, Tenon Hospital, University Pierre et Marie Curie, Paris, France

*Both authors contributed equally to this study. ^§^ Corresponding author:

Jaap D Legemate, M.D.

Department of Urology

AMC University Hospital

Meibergdreef 9

1105 AZ Amsterdam Z-O, The Netherlands

Tel: +31-20-5664377, Fax: +31-20-5669585

E-mail: [j.d.legemate@amc.uva.nl](mailto:j.d.legemate@amc.uva.nl)
